# 211. Disease burden of bacteremia with extended-spectrum beta-lactamase-producing or carbapenem-resistant Enterobacteriaceae in Korea

**DOI:** 10.1093/ofid/ofad500.284

**Published:** 2023-11-27

**Authors:** Chan Mi Lee, Shinwon Lee, Wan Beom Park, Pyoeng Gyun Choe, Chung-Jong Kim, Bongyoung Kim, Song Mi Moon, Yee Gyung Kwak, Yeon-Sook Kim, Young Keun Kim, Yu Jin Sohn, Kyung-Hwa Park, Seong Eun Kim, Eu Suk Kim, Hong Bin Kim, Jeonghoon Ahn, Kyoung-Ho Song

**Affiliations:** Seoul National University College of Medicine, Seoul, Seoul-t'ukpyolsi, Republic of Korea; Division of Infectious Disease, Department of Internal Medicine, Pusan National University Hospital, Seo-gu, Pusan-jikhalsi, Republic of Korea; Seoul National University College of Medicine, Seoul, Seoul-t'ukpyolsi, Republic of Korea; Seoul National University College of Medicine, Seoul, Seoul-t'ukpyolsi, Republic of Korea; Ewha Womans University College of Medicine, Seoul, Seoul-t'ukpyolsi, Republic of Korea; Department of Internal Medicine, Hanyang University College of Medicine, Seongdong-gu, Seoul-t'ukpyolsi, Republic of Korea; Seoul National University College of Medicine, Seoul, Seoul-t'ukpyolsi, Republic of Korea; Ilsan Paik Hospital, Ilsan, Kyonggi-do, Republic of Korea; Division of Infectious Diseases, Department of Internal Medicine, Chungnam National University School of Medicine, Daejeon, Taejon-jikhalsi, Republic of Korea; Yonsei University Wonju College of Medicine, Wonju, Kangwon-do, Republic of Korea; Wonju College of Medicine, Yonsei University, Wonju, Kangwon-do, Republic of Korea; Chonnam National University Medical School, GwangJu, Kwangju-jikhalsi, Republic of Korea; Chonnam National University Medical School, GwangJu, Kwangju-jikhalsi, Republic of Korea; Seoul National University Bundang Hospital, Bundang-gu, Kyonggi-do, Republic of Korea; Seoul National University College of Medicine, Seoul, Seoul-t'ukpyolsi, Republic of Korea; Ewha Womans University, Seoul, Seoul-t'ukpyolsi, Republic of Korea; Seoul National University College of Medicine, Seoul, Seoul-t'ukpyolsi, Republic of Korea

## Abstract

**Background:**

Despite the importance of multidrug-resistant organisms (MDRO) bacteremia, especially extended-spectrum β-lactamase-producing Enterobacteriaceae (ESBL-E) or carbapenem-resistant Enterobacteriaceae (CRE), the disease burden of those has not been investigated in detail. Thus, we tried to evaluate the clinical outcomes and estimate the socioeconomic burden of those MDROs bacteremia at a nationwide level in the Republic of Korea.

**Methods:**

We retrospectively searched for all cases of ESBL-E or CRE bacteremia, as well as matched control patients, in ten hospitals representing regions across the country over a six-month period. Patients with ESBL-E or CRE bacteremia were classified as the R group, while matched controls with antibiotic-susceptible bacteremia or those without infection were classified as the S and N groups, respectively. Patients’ clinical data, including demographic data, underlying medical conditions, and microbiologic data were collected. We estimated economic burden by considering medical expenses, loss of productivity, and total costs.

**Results:**

We identified a total of 795 patients, including 265 cases with ESBL-E or CRE bacteremia, and their matched controls after propensity-score matching. The mean total length of stay for ESBL-E and CRE matching in the R group were 7.8 days and 18.3 days longer compared to the S group, and 12.2 days and 14.5 days longer compared to the N group. The 90-day mortality rates for ESBL-E and CRE matching in the R, S, and N groups were 12.1%, 5.6%, 3.4%, and 24.0%, 12.0%, 0.0%, respectively. The total costs of the R, S, and N groups showed significant differences in both ESBL-E and CRE matching ($11,151 *vs.* $8,712 *vs.* $6,063, *P* = 0.004; $40,464 *vs.* $8,748 *vs.* $7,279, *P* = 0.024).

**Figure:**
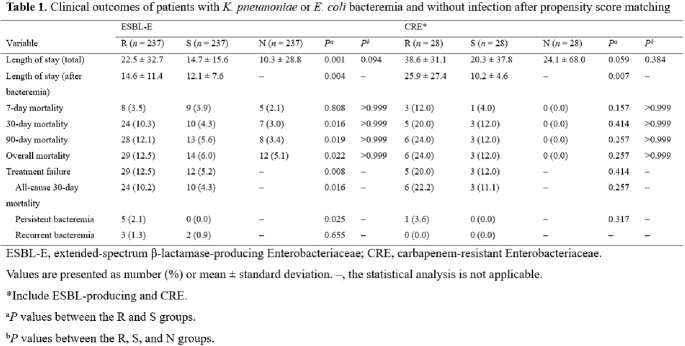

**Figure:**
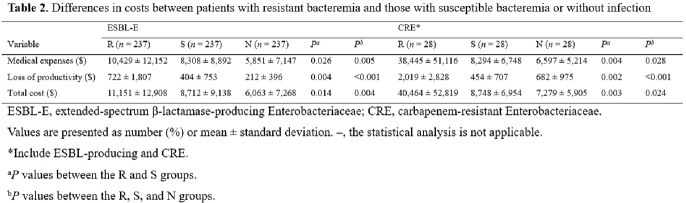

**Conclusion:**

The mortality rates of ESBL-E or CRE bacteremia were found to be tremendously high. Furthermore, the economic burden imposed by ESBL-E or CRE bacteremia, compared to controls without infection, were approximately two and five times higher, respectively. These findings suggest that ESBL-E and CRE bacteremia impose a significant clinical and economic burden, and adequate efforts to control these resistant bacteremia might be necessary to reduce this burden.

This Study is sponsored by Pfizer Pharmaceuticals Korea Ltd.

**Disclosures:**

**All Authors**: No reported disclosures

